# Drug-related problems and their determinants among stroke patients in suburban China: a PCNE-based analysis with targeted initiatives

**DOI:** 10.3389/fpubh.2026.1771680

**Published:** 2026-02-20

**Authors:** Ying Liu, Mingfen Wu, Jing Zhao, Wenhua Yang, Qi Wu, Li He, Sijie Zhang, Fengzhao Han

**Affiliations:** 1 Department of Pharmacy, Beijing Huairou Hospital, Capital Medical University Huairou Teaching Hospital, Beijing, China; 2 Department of Pharmacy, Beijing Tiantan Hospital, Capital Medical University, Beijing, China

**Keywords:** community pharmacy, drug adherence, drug-related problems, PCNE classification, stroke

## Abstract

**Background:**

Stroke remains a major public health burden in China, and the complexity of long-term pharmacotherapy often places stroke survivors at high risk for drug-related problems (DRPs). This study aimed to identify determinants of DRPs among community-dwelling stroke patients in suburban Beijing and to propose targeted initiatives for improving rational drug use.

**Methods:**

A cross-sectional study was conducted from August 2022 to December 2024 among 481 stroke patients. Pharmacists performed face-to-face interviews to collect demographic, clinical, and drug-related information. DRPs were identified and categorized using the Pharmaceutical Care Network Europe (PCNE) Classification System (V9.1). Chi-square tests and multivariate logistic regression were used to determine factors associated with DRPs.

**Results:**

A total of 482 DRPs were identified, corresponding to 575 cause entries. The most common problems involved suboptimal treatment effectiveness and issues requiring further clarification. Patient-related causes were predominant, especially intentional underuse or non-use of drug and inappropriate timing or dosing intervals. Logistic regression showed that alcohol consumption (OR = 1.770), comorbid diabetes (OR = 1.818), depression/anxiety symptoms (OR = 1.733), and higher animal fat intake (OR = 1.711) significantly increased DRP risk. Lower income, polypharmacy, and poorer self-rated health were also associated with DRPs.

**Conclusion:**

DRPs were highly prevalent among community-dwelling stroke survivors, driven largely by modifiable behavioral and drug-use factors. Integrating PCNE-based DRP identification with pharmacist-led community interventions may help optimize drug management, enhance treatment safety and effectiveness, and support long-term secondary prevention.

## Introduction

Stroke has long been recognized as the leading cause of death in China (1), and in recent years it has also become one of the major contributors to disability across both urban and rural regions. What makes the situation more concerning is that the incidence is still climbing year by year (2). As a disease characterized by high morbidity, high disability, high mortality, and a remarkable tendency to recur, stroke requires long-term and standardized secondary prevention. International and national guidelines (3–5) consistently emphasize that appropriate pharmacotherapy is one of the most effective ways to reduce the risk of recurrent stroke and to secure better clinical outcomes.

Yet, the reality in everyday clinical practice is far from simple. Many stroke survivors live with multiple comorbidities (6), and the accumulation of drugs over time easily leads to polypharmacy (7). Polypharmacy increases the likelihood of potentially inappropriate drug use and consequently raises the incidence of drug-related problems (DRPs) (8). A substantial body of literature (9–11) has shown that DRPs are not trivial, they are linked to increased morbidity, higher hospitalization and mortality rates, and escalating healthcare expenditures. For patients living in the community, where long-term management after stroke is critical, identifying and preventing DRPs becomes even more essential (12).

The Pharmaceutical Care Network Europe (PCNE) classification system provides a structured and internationally adopted framework for describing and analyzing DRPs (13). Applying this system to community-dwelling stroke patients allows healthcare providers to detect issues early, trace the underlying causes, and intervene more systematically.

In suburban districts such as those around Beijing, stroke survivors often rely heavily on community medical services and pharmacist follow-up. However, evidence on DRPs in this specific population remains scarce. Understanding which factors drive DRPs, and how these factors interact with patients’ drug behaviors, comorbidities, and psychological status could offer practical guidance for improving community-level pharmaceutical care.

Therefore, this study aimed to classify DRPs among stroke patients living in the community using the PCNE system and to explore the determinants associated with their occurrence. By mapping out the key contributors to DRPs, we hope to provide evidence-based recommendations that support pharmacist-led home drug services, enhance community drug management, and ultimately promote safe and rational drug use among stroke survivors in suburban of China.

## Materials and methods

### Study design and ethics

This study was conducted as part of the Capital’s Funds for Health Improvement and Research (Grant No. CFH2022-3-7141). We employed a cross-sectional design in the Huairou District of Beijing, with data collected from August 1, 2022 to December 31, 2024. The sample size was determined through a power analysis (90% power), with reference to similar community-based stroke studies regarding key outcome proportions (14), and convenience sampling was used to recruit participants. A total of 481 community-dwelling stroke patients completed the survey.

All participants provided informed consent before the interviews. Each face-to-face interview was carried out by qualified clinical pharmacists, lasted approximately 20 min, and involved an electronic questionnaire. To ensure consistency and minimize bias in the assessment of DRPs, all interviews and subsequent evaluations were conducted by clinical pharmacists who had obtained certification and possessed over 3 years of work experience. The evaluation process followed standardized criteria based on drug labels, clinical guidelines, and expert consensus. All DRP classifications were independently reviewed by a senior pharmacist. Any discrepancies were resolved through discussion by a dedicated quality control panel. The study protocol was reviewed and approved by the Ethics Committee of Beijing Huairou Hospital (Approval No. (2022)-(016)-02).

### Data collection

Inclusion criteria: (1) age≥18 years; (2) had experienced a stroke within the past 6 months and were not currently hospitalized; (3) used three or more prescribed drugs daily; (4) willing and able to participate in face-to-face interviews conducted by clinical pharmacists.

Procedure of visits and collaboration: The initial pharmacist visit was conducted face-to-face at various settings including hospitals, community clinics, or patients’ homes. Follow-up contacts were carried out via face-to-face meetings, phone calls, or WeChat as needed. For recruitment, some patients were referred to the study by their neurologists or family physicians after an assessment confirming they met the inclusion criteria. During the subsequent intervention phase, pharmacists communicated directly with the patients’ neurologists and/or family physicians to discuss and collaboratively adjust medication regimens.

Exclusion criteria: (1) patients with severe or terminal illnesses; (2) those with mental disorders or severe cognitive impairment; (3) inability to complete questionnaires; (4) incomplete clinical data precluding statistical analysis.

### Questionnaire content

The questionnaire was developed by a multidisciplinary expert group including pharmacists, clinicians, sociologists, and health management specialists. Its development process involved literature review, expert consultation, and multiple rounds of discussion. The final instrument consisted of two major sections: (1) Influencing Factors Questionnaire. Collected demographic information, health status, comorbidities, and drug-use history. (2) DRPs Assessment. DRPs were evaluated using the Pharmaceutical Care Network Europe (PCNE) Classification System (Version 9.1). The PCNE structure includes Problem (P), Cause (C), Planned intervention (I), Intervention acceptance (A) and Outcome (O). For the purposes of this study, only the first two components, problems and causes were analyzed. Pharmacists reviewed each case sequentially within the PCNE framework and selected corresponding classifications to achieve both qualitative and quantitative evaluation of DRPs.

### Data analysis

All analyses were performed using SPSS 26.0. Descriptive statistics were used to summarize participant characteristics. Chi-square tests were conducted to examine associations between the presence of DRPs and individual variables. Multiple logistic regression was then used to identify independent factors associated with DRPs. DRPs (yes/no) served as the dependent variable. Independent variables included marital status, education level, monthly income, monthly drug expenditure, medical reimbursement type, number of chronic diseases, comorbidities, lifestyle behaviors, health status indicators, disease-related factors, and history of adverse drug reactions. Gender and age were included as covariates. Variables for the multivariate logistic regression model were selected based on clinical relevance and a univariate screening threshold of *p* < 0.05. A sensitivity analysis using a threshold of *p* < 0.10 confirmed no additional variables warranted inclusion. The final model was built using the Enter method to include all preselected variables simultaneously.

## Results

### Participant characteristics

A total of 481 stroke patients were included in the analysis. Their basic demographic and clinical characteristics are summarized in Table 1. Among them, 295 (61.3%) were male and 186 (38.7%) were female. Most participants were aged 60 years or older (68.0%) and had a primary or junior high school education (68.6%). The majority reported a monthly income below 5,000 CNY.

**Table 1:** Distribution of basic characteristics of participants.

Characteristics			Total, *N*	DRPs, *N*		*χ* ^2^	*p*
				Yes	No		
Basic information	Gender	Male	295	166	129	1.805	0.179
		Female	186	93	93		
	Age	18–59, *n* (%)	154	86	68	0.364	0.546
		≥60, *n* (%)	327	173	154		
	Education level	Primary/Junior high school	330	180	150	0.695	0.706
		Senior high school or Junior college	117	63	54		
		Bachelor’s/Master’s degree	34	16	18		
	Marital status	Married	435	238	197	1.950	0.583
		Divorced	7	4	3		
		Widowed	36	16	20		
		Single	3	1	2		
Expenses	Monthly income, CNY	<5,000	393	203	190	4.154	0.042
		≥5,000	88	56	32		
	Monthly cost on drug, CNY	<500	354	186	168	0.931	0.628
		500–1,000	110	63	47		
		>1,000	17	10	7		
	Reimbursement type	Self-funded/other types	46	26	20	0.170	0.680
		Full or partial	435	232	203		
Living habits	Smoking	Yes	136	78	58	2.727	0.256
		No	247	124	123		
		Quit smoking	98	57	41		
	Alcohol consumption	Yes	140	88	52	6.452	0.011
		No	341	171	170		
	Tea consumption	Yes	274	141	133	1.459	0.227
		No	207	118	89		
	Coffee	Yes	54	33	21	1.292	0.256
		No	427	226	201		
	Regular exercise	Yes	372	200	172	0.005	0.946
		No	109	59	50		
Comorbidity	Hypertension	Yes	341	187	154	0.464	0.496
		No	140	72	68		
	With diabetes	Yes	165	105	60	9.686	0.002
		No	316	154	162		
	With hyperlipidemia	Yes	268	139	129	0.955	0.328
		No	213	120	93		
	With coronary heart disease	Yes	91	47	44	0.218	0.640
		No	390	212	178		
Eating habits	Intaking of animal fat	Yes	132	84	48	7.016	0.008
		Sometimes or no	349	175	174		
	Intaking of seafood	Yes	65	34	31	0.072	0.789
		Sometimes or no	416	225	191		
	Intaking of roughage	Yes	199	112	87	0.810	0.368
		Sometimes or no	282	147	135		
	Intaking of vegetables	Yes	319	171	148	0.022	0.882
		Sometimes or no	162	88	74		
	Intaking of nuts	Yes	67	36	31	0.000	0.984
		Sometimes or no	414	223	191		
	Taste	mild	192	108	84	0.743	0.389
		heavy	289	151	138		
Drug	Number of medicines	<5	139	59	80	10.223	0.001
		≥5	342	200	142		
Emotion	Depression and anxiety	Yes	88	57	31	5.174	0.023
		No	393	202	191		

Characteristics		Total, *N*	Total, IQR	With DRPs, IQR	Without DRPs, IQR	*Z*	*p*
Body mass	BMI (kg/m^2^)	481	25.34 (23.39, 27.48)	25.39 (23.44, 27.47)	24.80 (23.03, 27.51)	−1.091	0.275
Comorbidities	Number	481	3.00 (2.00, 4.00)	3.00 (2.00, 4.00)	3.00 (2.00, 4.00)	−1.632	0. 103
Quality of life	Self-assessed health score	481	70.00 (60.00, 80.00)	70.00 (60.00, 80.00)	70.00 (60.00, 85.00)	−2.540	0.011
	EQ-5D score	481	0.85 (0.77, 0.85)	0.85 (0.77, 0.85)	0.85 (0.77, 0.85)	−0.401	0.688

CNY, Chinese Yuan (¥); DRPs, drug-related problems; BMI, body mass index; IQR, interquartile range.

Regarding lifestyle behaviors, about one-third reported current smoking or alcohol consumption, and most participants engaged in some form of regular physical activity. Comorbidities were common, particularly hypertension (70.9%), hyperlipidemia (55.7%), and diabetes (34.3%). Nearly 30% reported high intake of animal fat.

Polypharmacy was prevalent, more than 70% used five or more drugs daily. Almost half of the participants exhibited poor drug adherence, and around 18% reported symptoms of depression or anxiety. Continuous variables showed that the average BMI fell within the overweight range, and self-assessed health scores were moderate.

Two hundred fifty-nine stroke patients were detected having DRPs. Chi-square analysis indicated that several factors including monthly income, alcohol use, comorbid diabetes, high animal fat intake, polypharmacy, depressive/anxiety symptoms, and lower self-assessed health scores were significantly associated with the presence of DRPs.

### Logistic regression analysis

Table 2 presents the multivariate logistic regression results. Several variables remained independently associated with DRPs after adjustment. Alcohol consumption significantly increased the likelihood of experiencing DRPs (OR = 1.770, *p* = 0.01). Comorbid diabetes was also an important determinant (OR = 1.818, *p* = 0.005). Higher animal fat intake (OR = 1.711, *p* = 0.016) significantly increased DRP risk. Depressive or anxiety symptoms were associated with the risk of DRPs (OR = 1.773, *p* = 0.037). Overall, the regression model suggests that behavioral, psychological, eating habits, and disease-related factors all contribute to DRP risk.

**Table 2:** Logistic regression analysis of factors associated with DRPs among stroke patients.

Variables		*β*	SE	Wald	*p*	OR	95%CI
Month income (ref = <5,000)	>5,000	0.278	0.274	1.093	0.296	1.332	0.778–2.281
Alcohol consumption (ref = No)	Yes	0.571	0.222	6.604	0.010	1.770	1.145–2.735
With diabetes (ref = No)	Yes	0.598	0.213	7.896	0.005	1.818	1.198–2.758
Intaking of animal fat (ref = sometimes or no)	Yes	0.537	0.224	5.758	0.016	1.711	1.103–2.652
Number of medicines (ref = <5)	>5	0.289	0.200	2.090	0.148	1.335	0.902–1.977
Depression and anxiety (ref = No)	Yes	0.550	0.264	4.332	0.037	1.733	1.033–2.909
Self-assessed health score		−0.01	0.006	3.344	0.067	0.990	0.979–1.001

### Assessment of DRPs and their causes

A total of 482 DRPs were identified in 259 patients according to the PCNE classification system (Table 3). The majority of DRPs were categorized under “other problems” (P3), primarily comprising unclear complaints or issues requiring further clarification (P3.2, *n* = 219). Problems related to treatment effectiveness were also frequent (P1; *n* = 197), with suboptimal treatment effect (P1.2; *n* = 188) being the most common subtype. A total of 52 treatment safety problems (P2.1) involved actual or potential adverse drug events.

**Table 3:** Distribution of DRPs and their causes based on the PCNE classification system.

DRPs	First-level directory		Second-level directory		Types of problems
	Code	*N*	Code	*N*	
Treatment effectiveness	P1	197	P1.1	4	No effect of drug treatment despite correct use
			P1.2	188	Effect of drug treatment not optimal
			P1.3	5	Untreated symptoms or indication
Treatment safety	P2	52	P2.1	52	Adverse drug event (possibly) occurring
Other	P3	233	P3.1	14	Unnecessary drug-treatment
			P3.2	219	Unclear problem/complaint. Further clarification necessary (please use as escape only)
Total		482		482	

Primary domain	First-level directory		Second-level directory		Causes
	Code	*N*	Code	*N*	
Prescribing and drug selection	C1	33	C1.1	10	Inappropriate drug according to guidelines/formulary
			C1.2	1	No indication for drug
			C1.3	0	Inappropriate combination of drugs, or drugs and herbal medications, or drugs and dietary supplements
			C1.4	3	Inappropriate duplication of therapeutic group or active ingredient
			C1.5	17	No or incomplete drug treatment in spite of existing indication
			C1.6	2	Too many different drugs/active ingredients prescribed for indication
	C2	7	C2.1	7	Inappropriate drug form/formulation (for this patient)
	C3	32	C3.1	21	Drug dose too low
			C3.2	3	Drug dose of a single active ingredient too high
			C3.3	3	Dosage regimen not frequent enough
			C3.4	0	Dosage regimen too frequent
			C3.5	5	Dose timing instructions wrong, unclear or missing
	C4	6	C4.1	3	Duration of treatment too short
			C4.2	3	Duration of treatment too long
Dispensing	C5	0	C5.1	0	Prescribed drug not available
			C5.2	0	Necessary information not provided or incorrect advice provided
			C5.3	0	Wrong drug, strength or dosage advised (OTC)
			C5.4	0	Wrong drug or strength dispensed
Use	C6	23	C6.1	9	Inappropriate timing of administration or dosing intervals by a health professional
			C6.2	11	Drug under-administered by a health professional
			C6.3	1	Drug over-administered by a health professional
			C6.4	2	Drug not administered at all by a health professional
			C6.5	0	Wrong drug administered by a health professional
			C6.6	0	Drug administered via wrong route by a health professional
	C7	330	C7.1	198	Patient intentionally uses/takes less drug than prescribed or does not take the drug at all for whatever reason
			C7.2	7	Patient uses/takes more drug than prescribed
			C7.3	0	Patient abuses drug (unregulated overuse)
			C7.4	4	Patient decides to use unnecessary drug
			C7.5	0	Patient takes food that interacts
			C7.6	2	Patient stores drug inappropriately
			C7.7	88	Inappropriate timing or dosing intervals
			C7.8	18	Patient unintentionally administers/uses the drug in a wrong way
			C7.9	11	Patient physically unable to use drug/form as directed
			C7.10	2	Patient unable to understand instructions properly
Seamless	C8	1	C8.1	1	Medication reconciliation problem
Other	C9	143	C9.1	2	No or inappropriate outcome monitoring (incl. TDM)
			C9.2	138	Other cause; specify
			C9.3	3	No obvious cause
Total		575		575	

Regarding causes, 482 DRPs can be attributed to 575 causes. Patient-related factors (C7) were the dominant category (*n* = 330). The most common issues included intentional underuse or non-use of drugs (C7.1; *n* = 198) and inappropriate timing or dosing intervals (C7.7; *n* = 88). Other notable causes included dosage-related problems (C3; *n* = 32), prescribing or drug selection issues (C1; *n* = 33), administration errors by healthcare professionals (C6; *n* = 23), and inadequate outcome monitoring (C9; *n* = 143).

Together, these findings indicate that DRPs among community-dwelling stroke patients primarily arise from suboptimal treatment effectiveness and patient-driven behaviors, particularly non-adherence and improper drug use.

### Distribution of DRPs by drug category

**Figure 1:**
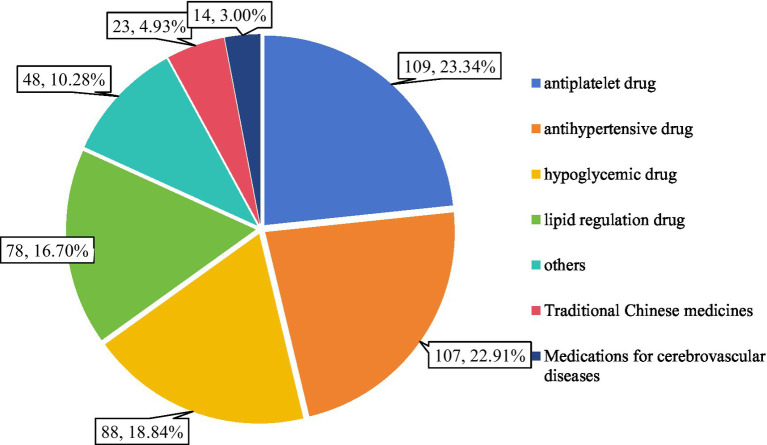
Distribution of drug categories associated with identified DRPs among stroke patients.

**Figure 2:**
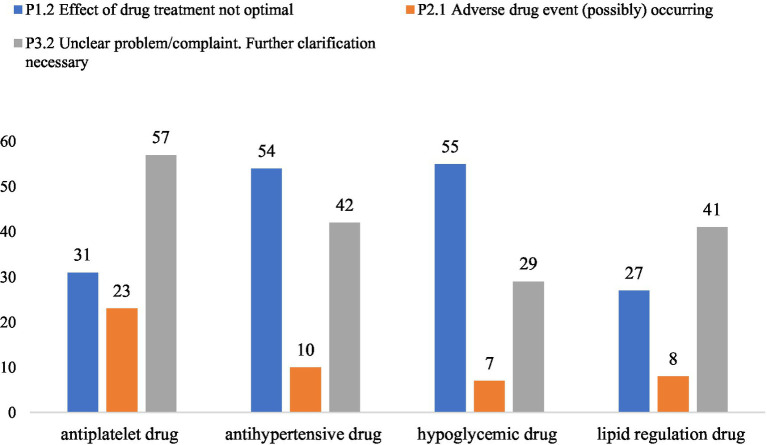
The four drug categories with the highest number of DRPs.

Figure 1 shows the distribution of 482 DRPs across drug categories. The top four drugs most frequently associated with DRPs were antiplatelet agents, antihypertensives, antidiabetic drugs, and lipid-lowering drugs. The four drug categories with the highest number of DRPs are exhibited in Figure 2. For antiplatelet and lipid-lowering agents, the most common DRP type was “unclear problem/complaint,” often reflecting concerns requiring further clarification. For antihypertensives and antidiabetic agents, “suboptimal treatment effect” was the dominant issue.

**Figure 3:**
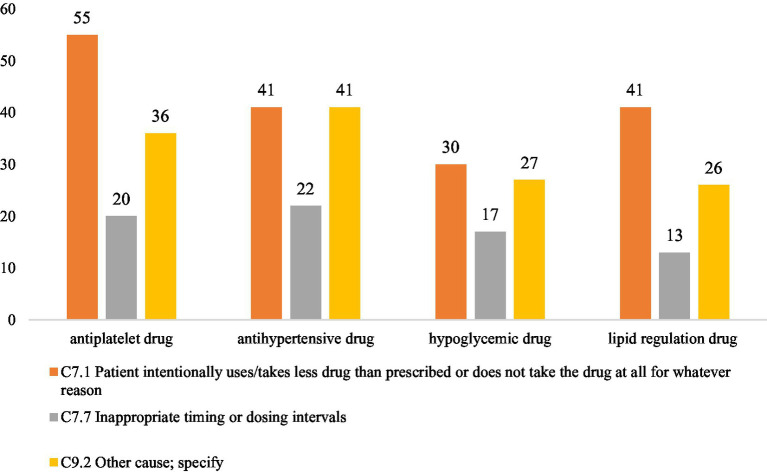
Distribution of PCNE-classified causes for DRPs in the top four drug categories.

Figure 3 shows the distribution of PCNE-classified causes for DRPs in the top four drug categories. Across all four categories, the leading cause was intentional underuse or discontinuation (C7.1), followed by “other causes” (C9.2) and inappropriate timing or dosing intervals (C7.7). A deeper examination suggested that antihypertensive and antidiabetic problems often emerged when blood pressure or blood glucose levels remained uncontrolled. In contrast, DRPs related to antiplatelet and lipid-lowering drugs frequently stemmed from patients discontinuing therapy due to concerns about potential adverse effects, thereby increasing their risk of recurrent ischemic events.

Table 4 shows the distribution of active ingredient of four drug categories with the highest number of DRPs. In the analysis of the four types of drugs with a relatively high incidence of DRPS, antiplatelet drugs are mainly aspirin, antihypertensive drugs are mainly CCBs and ARBs, hypoglycemic drugs are mainly biguanides, alpha-glucosidase inhibitors and insulin, and lipid-regulating drugs are mainly statins. The causes of occurrence are consistent with the overall distribution.

**Table 4:** The distribution of active ingredient of four drug categories with the highest number of DRPs.

	Code	Antiplatelet drug			Antihypertensive drug						Hypoglycemic drug								Lipid regulation drug		
		Total	Aspirin	Clopidogrel	Total	CCB	ARB	Compound preparation	*β* blockers	Others	Total	Biguanides	*α*-glucosidase	Insulin and its analogues	SGLT-2i	DDP4 inhibitor	Sulfonylureas	Ohers	Total	Statins	Antioxidant class
P1	P1.1	2	1	1	1	1													1	1	
	P1.2	31	27	4	54	31	14	3	3	3	55	25	10	8	3	3	2	4	27	27	
	P1.3				1		1				1	1							1		
P2	P2.1	23	21	2	10	7		2	1		7	3		2	1		1		8	7	1
P3	P3.1				2	1	1				1	1							1	1	1
	P3.2	57	52	5	42	21	13	4	3	1	29	17	6		2	1	1	2	41	41	
Total		113			110						93								79		
C1	C1.1				3	1	1	1			2	1					1				
	C1.4				3	3															
	C1.5										12	5	2	3			1	1	2	2	
C2	C2.1	2	1	1	3				1	2									1	1	
C3	C3.1				7	3	3	1			14	6	4	4							
	C3.2				3	2		1													
	C3.3				1		1				2	1	1								
	C3.5	3	3		1	1													1	1	
C4	C4.1										2				1	1					
C6	C6.1	2	2		1	1					4	3						1	1	1	
	C6.2				1	1					9	3	3	2		1			1	1	
	C6.3				1	1															
	C6.4	1		1																	
C7	C7.1	55	50	5	41	23	12	3	3		30	18	4		2	2	3	1	41	41	
	C7.2	1	1		1	1					3	1		2							
	C7.4																		1	1	
	C7.6										2	1						1			
	C7.7	20	17	3	22	16	5			1	17	10	4					3	13	12	1
	C7.8	6	6		2	1	1				5	2	2		1						
	C7.9	4	3	1	3				1	2									2	2	
	C7.10																		1	1	
C9	C9.1				1	1															
	C9.2	36	30	6	41	21	11	4	3	2	27	14	5	2	1	1	1	3	26	25	1
	C9.3	1	1		2			1	1												
Total		131			137						129								90		

### Targeted initiatives for improving rational drug use

Based on the major determinants identified in this study, such as alcohol consumption, comorbid diabetes, higher animal fat intake, depressive or anxiety symptoms, and polypharmacy, as well as the distribution of PCNE-classified DRPs, several focused and feasible intervention strategies are suggested for community settings.

1. Strengthen drug adherence support. Our study found that poor drug adherence is a common type of DRPs in stroke patients. Implement structured pharmacist follow-up through home visits, phone calls, or digital reminders. When possible, simplify drug regimens and provide clear, user-friendly instructions to help patients take their drugs correctly.
2. Address alcohol-related risks. Offer brief, evidence-based counselling on the impact of alcohol on stroke recurrence and drug effectiveness. Develop stepwise reduction plans and link patients to psychological or behavioral support when necessary.
3. Optimize drug management for patients with diabetes. Encourage regular self-monitoring of blood glucose and ensure pharmacist involvement in adjusting therapy. Monitor for potential interactions between antidiabetic and antithrombotic agents and assess hypoglycemia risk.
4. Provide psychological screening and support. Routinely screen for depression and anxiety using validated tools. Establish referral pathways for mental health services and closely monitor the safety and effectiveness of antidepressant therapy where applicable.
5. Improve polypharmacy management. Conduct regular pharmacist-led drug reviews to identify therapeutic duplication, unnecessary drugs, and inappropriate dosages. Strengthen communication among healthcare providers to reduce inconsistent prescribing.
6. Promote healthy lifestyle and dietary habits. Encourage dietary adjustments, such as reducing animal fat intake and following heart-healthy eating patterns, and provide clear guidance regarding food–drug interactions.
7. Enhance community-based pharmaceutical care. Expand pharmacist-led services such as home drug reviews, chronic disease management clinics, and digital drug management systems to create a continuous and traceable model of care.

Overall, these targeted strategies support the development of a pharmacist-centered, closed-loop drug management system aimed at reducing DRPs and improving therapeutic outcomes among community-dwelling stroke patients.

## Discussion

This study provides a detailed examination of DRPs among community-dwelling stroke patients in suburban Beijing and shows that DRPs are not only common but also closely tied to patient behavior, drug management practices, and psychological factors. Using the PCNE classification system, we observed that patient-related causes, especially intentional underuse, non-use, and timing errors, far exceeded prescriber- or system-related causes. This pattern is consistent with previous research showing that stroke survivors often struggle with long-term drug self-management and maintaining adherence over time (15–17).

One notable finding was the high proportion of DRPs categorized as **“**Unclear problem/complaint” (P3.2, 45%). This reflects a significant real-world challenge in post-stroke care management. Our analysis suggests that this category was predominantly composed of cases where patients intentionally discontinued essential secondary prevention medications (e.g., aspirin, atorvastatin), citing a belief that these drugs were unnecessary or ineffective for their condition. This perception constitutes a critical gap in patient understanding of the long-term, preventive benefits of such therapies, which are often not immediately tangible. The high frequency of P3.2 classifications underscores that in clinical practice, a major category of drug-related “problems” is not a pharmacological issue, but rather a knowledge and adherence barrier rooted in patient health beliefs (18). This finding powerfully reinforces the indispensable role of clinical pharmacists in moving beyond simple medication review to providing tailored, persistent patient education. Future interventions should focus on improving patients’ perception of treatment necessity and long-term benefit to address this fundamental cause of non-adherence (19).

Almost half of the participants exhibited poor drug adherence, this finding echoes robust evidence from large clinical registries and cohort studies demonstrating that greater adherence to secondary prevention drugs significantly reduces mortality and improves long-term outcomes after stroke or transient ischemic attack (20). A meta-analysis of post-stroke adherence also showed that non-adherence remains a widespread challenge, with adherence declining markedly over the first year after stroke (16). These data support the present study’s result, highlighting the need for structured, pharmacist-led interventions, such as pharmacist follow-up, regimen simplification, and digital adherence tools to support long-term drug persistence.

Behavioral factors, including alcohol consumption, also contributed significantly to DRPs. Daily alcohol consumption is directly and closely associated with the occurrence of DRPs. For some patients with chronic conditions, drinking may exacerbate or destabilize their illnesses, such as hypertension, gastric ulcers, and gout (21). Additionally, under the influence of alcohol, patients may exhibit behaviors like forgetting to take medication or taking duplicate doses. Therefore, for patients with chronic diseases and those on long-term medication, pharmacists educating them to abstain from or strictly limit alcohol is a crucial guarantee for safe and effective treatment. High-fat dietary habits have a clear and significant association with the occurrence of drug-related problems (DRPs) (*p* < 0.05). Such habits can induce or exacerbate underlying conditions such as hypertension and hyperlipidemia (22), complicating pharmacotherapy, while also interfering with the absorption of certain medications. Therefore, for patients requiring long-term medication, especially those with chronic diseases, maintaining a balanced diet and controlling fat intake is not only essential for disease management but also serves as a fundamental measure to ensure medication safety and reduce the risk of DRPs.

Comorbid diabetes further complicated drug regimens. Diabetic patients often take multiple long-term drugs and are more susceptible to drug–drug interactions, hypoglycemia risk, and increased treatment burden, as documented in diabetes-related polypharmacy studies (23). These findings reinforce the importance of integrated chronic care and pharmacist involvement for patients with multiple comorbid conditions.

Psychological conditions, particularly depression and anxiety were identified as additional drivers of DRPs. Post-stroke affective disorders are known to impair motivation, reduce engagement in health behaviors, and negatively affect drug-taking routines. Previous meta-analyses found that depression after stroke is associated with poorer adherence and worse functional outcomes (24, 25). Our findings align with this evidence, suggesting that screening for depression and anxiety and offering timely mental health support should be essential components of community-based stroke management.

Notably, we found that income remained statistically insignificant. We speculate that this may be due to China’s healthcare insurance policies and centralized procurement measures, which keep patients’ drug costs low enough to be covered by their monthly income. As a result, low income does not have a significant impact on the incidence of DRPs among patients.

Taken together, this study emphasizes that DRPs among stroke survivors are largely preventable and driven by modifiable patient-related factors. Previous work has shown that chronic disease management programs, particularly those involving pharmacists, can improve long-term drug adherence, simplify regimens, and reduce drug errors (26). The present findings support adopting similar models in community settings, where pharmacists play a central role in counseling, monitoring outcomes, performing drug reviews, and addressing patients’ concerns about their therapy.

The cause analysis using PCNE further demonstrated that DRPs were predominantly patient-related rather than prescriber-related. While some dosage or prescribing issues were present, patient-driven causes far outnumbered them, echoing previous studies in community and chronic disease populations (12, 27). These results underscore the need for pharmacist-led interventions, such as home drug reviews, simplified dosing schedules, clear communication through pictorial instructions, and outcome monitoring to reduce preventable DRPs.

### Limitations

Our study still has some limitations. First, it was conducted in a single suburban district, which may limit generalizability. Second, data on adherence and lifestyle behaviors were self-reported, possibly introducing bias. Thirdly, we employed a convenience sampling method, with patients primarily sourced from hospital outpatient departments, referrals by neurologists, and recommendations from family physicians. As a result, data from bedridden patients or those unable to attend hospital visits were not comprehensively collected, which constitutes a limitation of this study. Future studies could employ longitudinal designs, objective adherence measures, and multi-site recruitment to validate and extend these findings.

## Conclusion

DRPs are highly prevalent among community-dwelling stroke survivors in suburban Beijing. Their occurrence is strongly influenced by behavioral and lifestyle factors, comorbidities, and psychological status. Patient-related causes, especially intentional underuse and inappropriate drug timing dominated the PCNE cause spectrum. A pharmacist-centered, community-based care model that integrates DRP identification, personalized counseling, adherence support, and coordinated chronic disease management holds promise for reducing DRPs and improving therapeutic outcomes. Implementing such interventions may ultimately strengthen secondary prevention and reduce the long-term burden of stroke.

## Data Availability

The original contributions presented in the study are included in the article/supplementary material, further inquiries can be directed to the corresponding author.
